# Development of a hepatocellular carcinoma imaging database and structured imaging reports based on PACS, HIS, and repository

**DOI:** 10.3389/fonc.2022.1033478

**Published:** 2023-02-16

**Authors:** Yushu Shi, Yufeng Tao, Jing Li, Zhi Li, Rui Zhang, Feng Chen

**Affiliations:** ^1^ First Affiliated Hospital, School of Medicine, Zhejiang University, Hangzhou, Zhejiang, China; ^2^ Second Affiliated Hospital, School of Medicine, Zhejiang University, Hangzhou, Zhejiang, China

**Keywords:** hepatocellular carcinoma, intelligent diagnosis and treatment, database, C/S mode, SQL server

## Abstract

**Purpose:**

To establish a hepatocellular carcinoma imaging database and structured imaging reports based on PACS, HIS, and repository.

**Methods:**

This study was approved by the Institutional Review Board. The steps of establishing the database are as follows: 1) According to the standards required for the intelligent diagnosis of HCC, it was attempted to design the corresponding functional modules after analyzing the requirements; 2) Based on client/server (C/S) mode, 3-tier architecture model was adopted. A user interface (UI) could receive data entered by users and show handled data. Business logic layer (BLL) could process the business logic of the data, and data access layer (DAL) could save the data in the database. The storage and management of HCC imaging data could be realized by the SQLSERVER database management software, and Delphi and VC++ programming languages were used.

**Results:**

The test results showed that the proposed database could swiftly obtain the pathological, clinical, and imaging data of HCC from the picture archiving and communication system (PACS) and hospital information system (HIS), and perform data storage and visualization of structured imaging reports. According to the HCC imaging data, liver imaging reporting and data system (LI-RADS) assessment, standardized staging, and intelligent imaging analysis were carried out on the high-risk population to establish a one-stop imaging evaluation platform for HCC, strongly supporting clinicians in the diagnosis and treatment of HCC.

**Conclusions:**

The establishment of a HCC imaging database can not only provide a huge amount of imaging data for the basic and clinical research on HCC, but also facilitate the scientific management and quantitative assessment of HCC. Besides, a HCC imaging database is advantageous for personalized treatment and follow-up of HCC patients.

## 1 Introduction

Hepatocellular carcinoma (HCC) is one of the most common malignant tumors in the world, as well as being the fourth most common malignant tumor and the third leading cause of cancer-related death in China (1). To date, several studies have applied deep learning, neural network, radiomics, and other artificial intelligence (AI) methods to imaging diagnostic evaluation and prognostic prediction of HCC patients *via* extracting and analyzing the clinical data (2–4). Thus, it is of great significance to concentrate on the occurrence, progression, and prognosis of HCC for the precise diagnosis and treatment of HCC. However, the single-center nature of the majority of these studies hindered generalizability of their findings. In spite of progresses achieved in data mining and performing large sample size research, there is no public HCC database worldwide. The establishment of a HCC imaging database can provide a huge amount of data for clinical research, which can not only meet the needs of their own research, but also share resources with researchers from other hospitals, research institutions, and universities. As a result, a comprehensive HCC imaging database is beneficial to the scientific management and quantitative assessment of HCC, and to perform personalized treatment and follow-up of HCC patients (5).

The repository refers to the intelligent knowledge system (or expert system) by the combination of two computer technologies, including AI database (6). Therefore, a database is the basis of repository, and repository is an upgraded version of the database. A repository is also different from the general application program, because the general application program implicitly encodes the knowledge of solving problem, while the repository shows the knowledge explicitly, and forms a relatively independent program entity. The essence of the repository is to develop a computer model, enabling experts to solve the problem (7), and the completeness and accuracy of the data in the repository directly affect the clinical decision-making.

Because of its advantages of simplicity, accuracy, and clinical relevance, structured imaging reports have been widely applied in different organs, such as prostate, lung cancer, breast, etc. (8–10). However, to date, the structured imaging report of HCC has not yet been presented in China. The clinical imaging report of HCC is basically descriptive, and the format and content of reports from various sources are different, hindering its applicability in clinical decision-making. Therefore, there is an urgent need to develop structured imaging reports of HCC.

The present study aimed to establish an imaging database of HCC based on picture archiving and communication system (PACS), hospital information system (HIS), and repository. It was attempted to design the corresponding functional modules after analyzing the requirements, adopt C/S (client/server) mode and a three-tier architecture mode (user interface (UI), business logic layer (BLL), and data access layer (DAL)), use SQL server management software to realize data storage and management, apply the intelligent knowledge system to the imaging diagnosis of HCC, and utilize the database information to initially create the structured imaging reports of HCC. Finally, a HCC data service platform based on PACS, HIS and repository, could be successfully established for collection, retrieval, and analysis of HCC data.

## 2 Materials and methods

### 2.1 Design of HCC imaging database

Based on semantic technology, the platform combines database, repository, and AI to establish a HCC imaging database based on PACS, HIS and repository. Besides, the domain knowledge in the repository is integrated with patients’ electronic medical records (EMRs) and PACS to realize the combination of multi-dimensional and multi-level knowledge and standardized expression of data, providing an effective decision support for the selection and adjustment of clinical treatment plans.

### 2.2 Functional design

Semantic technology is a concept in computer science that aims to bring semantics (the meaning and context of words and sentences) to the computer world, in simple terms, to allow computers to understand words and the meaning aiming to improve computer analysis and ability to understand language. Based on semantic technology, the database can be designed using three modules, including data layer, knowledge layer, and application layer. The data layer mainly concentrates on data retrieval, data collection, and data processing. Data retrieval and data collection include extracting patients’ basic information, examination of information, and diagnosis and treatment information from EMRs, as well as collecting daily real-time HCC imaging data confirmed by surgery and pathology according to subject headings. The knowledge layer is consisted of relevant content in the repository, including clinical guidelines, medical literature, experts’ experience and related professional knowledge, HCC staging and classification, structured imaging reports, and processed data. The application layer includes data processing and analysis, statistical analysis, and clinical decision-making.

**Figure 1:**
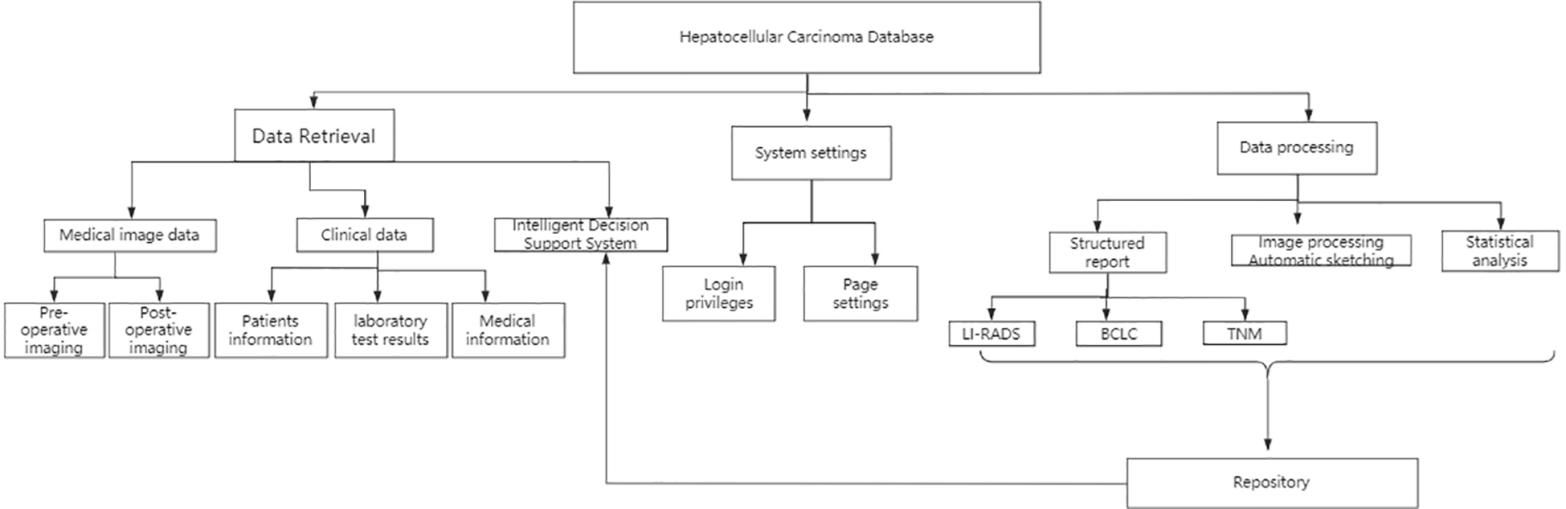
Flow chart and function module of HCC image database.

The proposed platform takes computed tomography/magnetic resonance imaging (CT/MRI) of liver as the core and incorporates clinically relevant data, including patients’ basic data, examination-related data, and diagnostic and therapeutic data, providing a comprehensive database for the accurate diagnosis and structured imaging reports of HCC. The structured imaging report should include the following contents: quantitative analysis of tumor, liver imaging reporting and data system (LI-RADS) (11), HCC staging (12), treatment method (13–16), and other significant information. The DICOM C-MOVE command was utilized in the PACS system, which automatically collects, archives, permanently online stores the original images and diagnostic reports of pathologically confirmed HCC cases in real-time, and automatically retrieves clinically relevant data and sync matching and archiving. The overall functional design of the system is shown in **Figure 1**, mainly consisting of three main modules of data retrieval, system setting, and data processing, and further subdivides each sub-module according to the function of the parent node.

### 2.3 Structural design

**Figure 2:**
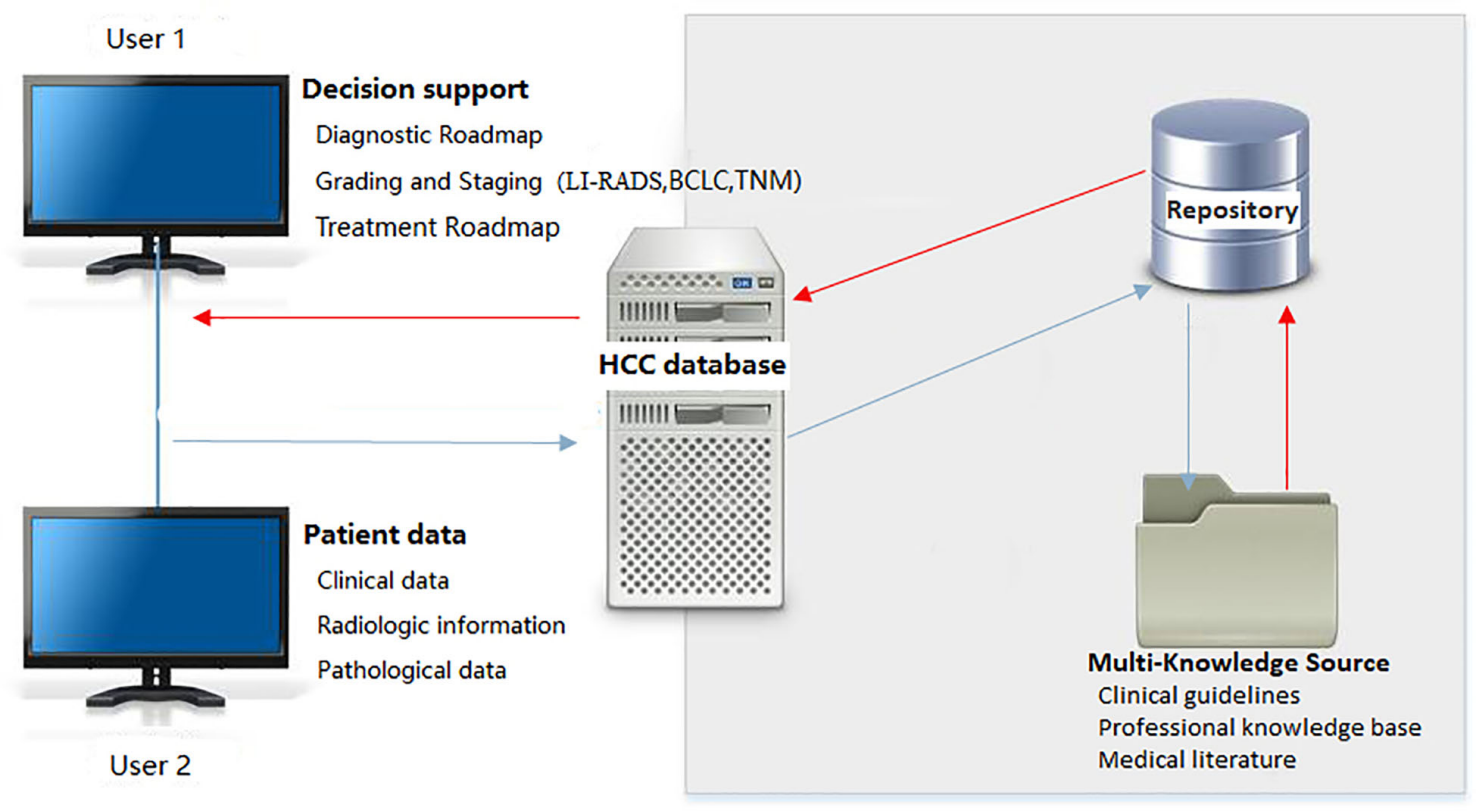
System architecture of HCC image database. LI-RADS, Liver Imaging Reporting and Data System; BCLC, Barcelona Clinic Liver Cancer; TNM, Tumor Node Metastasis classification.

The database is divided into two parts: data retrieval in the foreground and data management in the background. When a user logs into the software interface, data retrieval is performed in the foreground, and after retrieving the relevant data, it is connected to the background data for processing (**Figure 2**). The foreground interface design mainly includes various functions, such as imaging data query, pathological diagnosis query, inspection index query, comprehensive query, and decision query. The imaging data query comprises multiple and various imaging examination queries of the same patient. While browsing the image data, routine operations on the image (i.e., window width bed adjustment, image roaming, image registration, and preset window operations) can be performed. A tool can also be used for intelligent area measurement (automatic or manual delineation), quantitative evaluation, etc. After post-processing of annotated images, the original images are not overwritten, and are separately uploaded onto the same case sub-directory to ensure the security of the original imaging data. At the same time, each account corresponds to the personal data under the same case list to ensure personalized storage of the data. The pathological diagnosis query can be searched according to the search term, HCC, and the specified subject words or/and their combinations; the inspection index query can be searched according to the specified search terms, such as alpha-fetoprotein (AFP), CA199, carcinoembryonic antigen (CEA), etc. In the comprehensive query, query can be carried out according to a patient’s ID number, gender, age, and date of admission and discharge. Decision query mainly aims to query the clinical decision suggestions made after the data processing by the knowledge layer. The front-end interface is also equipped with help and message functions, making it convenient for users to inquire about the basic operation steps and leave a message for disposal after finding a problem.

Data management in the background includes storage of clinical and inspection-related data, storage of intelligent imaging data, export of clinical and imaging data as Excel format, etc. This part mainly involves data storage and security, which requires the authority to manage the account. Ordinary users only have the authority to read data and upload and process data, so as to ensure the security and stability of the data. In order to ensure data security and prevent the loss of important data, all data are stored online on the PACS in the hospital. The original data can only be read and downloaded and cannot be changed. The processed data are stored independently under the same directory and sub-account and are invisible between sub-accounts to ensure the confidentiality of personalized data.

### 2.4 Database design

According to the standardized design method, the whole process of developing the database and the application system needs to be considered. The database is not only a simple storage of images and clinical data, but also needs to consider the conceptual design. The present study adopts an E-R model (entity-relationship diagram), a logical design (ER model to relational model), and physical design of database (storage structure and access path of database).

Conceptual design is the first stage in the database design process. After analyzing entities in the database, it summarizes and abstracts the attributes of each entity and the relationship between each pair of entities, forming an E-R model that is independent of the specific database management system (DBMS). The ER model of the HCC imaging database includes a total of 7 entities, including patients’ basic information, pathological diagnostic report, CT/MRI report, examination report (tumor marker), CT/MRI path table, treatment method, and postoperative follow-up data (**Table 1**). The logical design phase (E-R diagram) aims to convert the conceptual design into a data model supported by DBMS and to optimize it.

**Table 1:** Logic Design of HCC database.

Data source	Field 1	Field 2	Field 3	Field 4	Field 5
Patient information & pathologic diagnosis	SN PK, int ()	Age tinyint ()	Hepatitis History varchar ()	Pathology text	
HCC Data	SN PK, int ()	Size/diameter int ()	Type varchar ()	Patient SN FK, int ()	
CT/MR Data	SN PK, int ()	Scan time int ()	Radiologic Report text	Lesion Site text	
Tumor markers	SN PK, int ()	AFP decimal ()	CA-199 decimal ()	CA125 decimal ()	
CT/MR Roadmap	SN PK, int ()	Thumbnail path char ()	Creation Time varchar()	Patient SN FK, int ()	
Form of treatment	SN PK, int ()	Surgical Resection varchar ()	TACE+ SR varchar ()	TACE varchar ()	Other varchar ()
Follow up data	SN PK, int ()	Survival Period tiny ()	Death varchar ()	Note tinytext	

SN, Serial Number; TACE, Transcatheter Arterial Chemoembolization; SR, Surgical resection; AFP, Alpha-fetoprotein.

There are some retrieve the value of fields and script codes in our software.

There are three main types of conflicts between parts of the E-R diagram: attribute conflicts, naming conflicts, and structural conflicts. In the conversion of E-R diagram to a relational model, it is necessary to indicate how to convert the entity and the relationship between entities into relational schemas, and how to determine the attributes and codes of these relational schemas. For instance, the relationship between patient and lesion pathological data, imaging data, and tumor markers is 1-to-1, the relationship between imaging data and patient, imaging data and HCC is many-to-1, and the relationship between pathological data and imaging data is many-to-many. The above-mentioned problem can be solved through a logical design, and database administration is therefore necessary. Physical design mainly refers to the database storage and storage path, that is, the logical design of the database is implemented using an actual physical storage device, so as to establish a physical database with a better performance. In the physical design stage, indexes should be established on the relevant attributes or combinations of attributes according to the requirements of index optimization to optimize the physical structure of the database. Additionally, a database is a system shared by multiple users, and multiple access paths must be established for the same relationship to meet the multiple application requirements of multiple users. One of the tasks of physical design is to determine which access method should be selected. There are three common access methods, including index method, cluster method, and HASH method. The B+ tree index method in the index method has been widely used because of its high efficiency. Thus, we used the B+ tree index method in the proposed database.

### 2.5 Implementation technique

#### 2.5.1 Development plan

The proposed database is based on the design pattern of C/S. The SQL 2008R2 (relational database management system) server was used, and its functions include storage and backup of HCC imaging data. A client can adopt the framework of ASP+Flexbox+HTML5 and programming languages of Delphi and VC++, and its function includes data storage, visualization, query and writing structured imaging reports.

In the C/S three-tier architecture of the database, the UI receives and displays HCC data according to the type of tags; BLL processes the business logic of the data, including data validation and calculation; DAL saves the data processed by BLL in the database. The advantage of using the three-layer architecture pattern is to reduce the dependency between layers and to realize the idea of “high cohesion, low coupling”, which is conducive to standardization and reuse of logic at each layer. In addition, the present study adopts the C/S architecture, mainly because the system is used in the internal network of the hospital. Using the C/S architecture can complete the data processing on the client side, the response speed is faster, and it is easier to realize the complex logic of the structured imaging report. Moreover, individual requirements, such as structured reporting system and staging and grading are changeable, and the use of C/S architecture can promptly realize individualized configuration of UI and operational habits.

#### 2.5.2 Key technology

Using the three-tier architecture, it is necessary to develop the database system for the three layers of UI, BLL, and DAL. First, UI is located in the outermost layer (the top layer), facing the user, using Active Server Pages (ASP), HyperText Markup Language (HTML), and Adobe Flex (FLEX) technology for front-end interface development and design, aiming to provide an interactive interface for users. BLL reflects the core value of the system architecture, which is located in the middle of UI and DAL, and serves as the link between the previous and the next stages in the data exchange. This layer is realized by the Active Template Library (ATL) of VC++. Visual C++ is an integrated development environment used to create Windows applications in the C, C++, and C++/CLI programming languages. ATL is a Microsoft program library that supports the use of C++ language to write ASP code and other ActiveX programs. Through the active template library, users can build COM components, and then call the COM objects through the script in the ASP page. The DAL, also known as the persistence layer, is mainly responsible for database access, as well as storage and management of relevant data. DAL uses MySQL technology and is a relational database management system, which is widely used due to the advantages of open source, excellent performance, and great portability. Users of this system can set specific labels, such as HCC, focal liver nodular hyperplasia (FNH), liver nodular regenerative hyperplasia (NRH), cholangiocarcinoma (ICC), etc., and then the program will automatically search relevant pathological, imaging, and clinical data (the service supports data interaction through various interfaces, such as database intermediate tables, WebService, and Health Level Seven (HL7, a standard for medical informatics exchange between healthcare providers)) in the RIS system. Then, it automatically collects, organizes, and summarizes data on the server, and all files are available in the PACS and HIS in the hospital. Users can perform secondary analysis on the retrieved data, save structured imaging reports, and export to the Excel. DICOM images can be obtained from PACS through DICOM Query/Retrieve, and images and data can be anonymized.

#### 2.5.3 Quantitative assessment, LI-RADS assessment, and treatment route in the repository

After data collection, the software performs quantitative and LI-RADS assessments of tumors, which can be convenient for further research and analysis. Quantitative assessment is mainly utilized for objective evaluation of target tumors and subjective evaluation of non-target tumors. Size, location, CT value, enhancement mode, peripheral invasion, and lymph node metastasis of target tumors are quantified. All measurement and quantification points are assessed and the current measurement tasks are submitted and reports are created. After entering the LI-RADS evaluation interface, if the current lesion organ is associated with multiple measurement trajectory values, users should enter the classification evaluation wizard. If the current lesion has only one measurement trajectory value, users can skip this step and directly perform LI-RADS assessment and then create LI-RADS reports. After the quantitative and LI-RADS assessments, users should return to the main page and perform HCC staging assessment based on the grading assessment. The repository contains clinical guidelines, medical literature, and specialized repository. Through preliminary quantitative assessment, LI-RADS assessment, and staging and grading, AI can be used to analyze the relevant data in the repository, and some effective treatment measures and guidance can be presented.

## 3 Results

**Figure 3:**
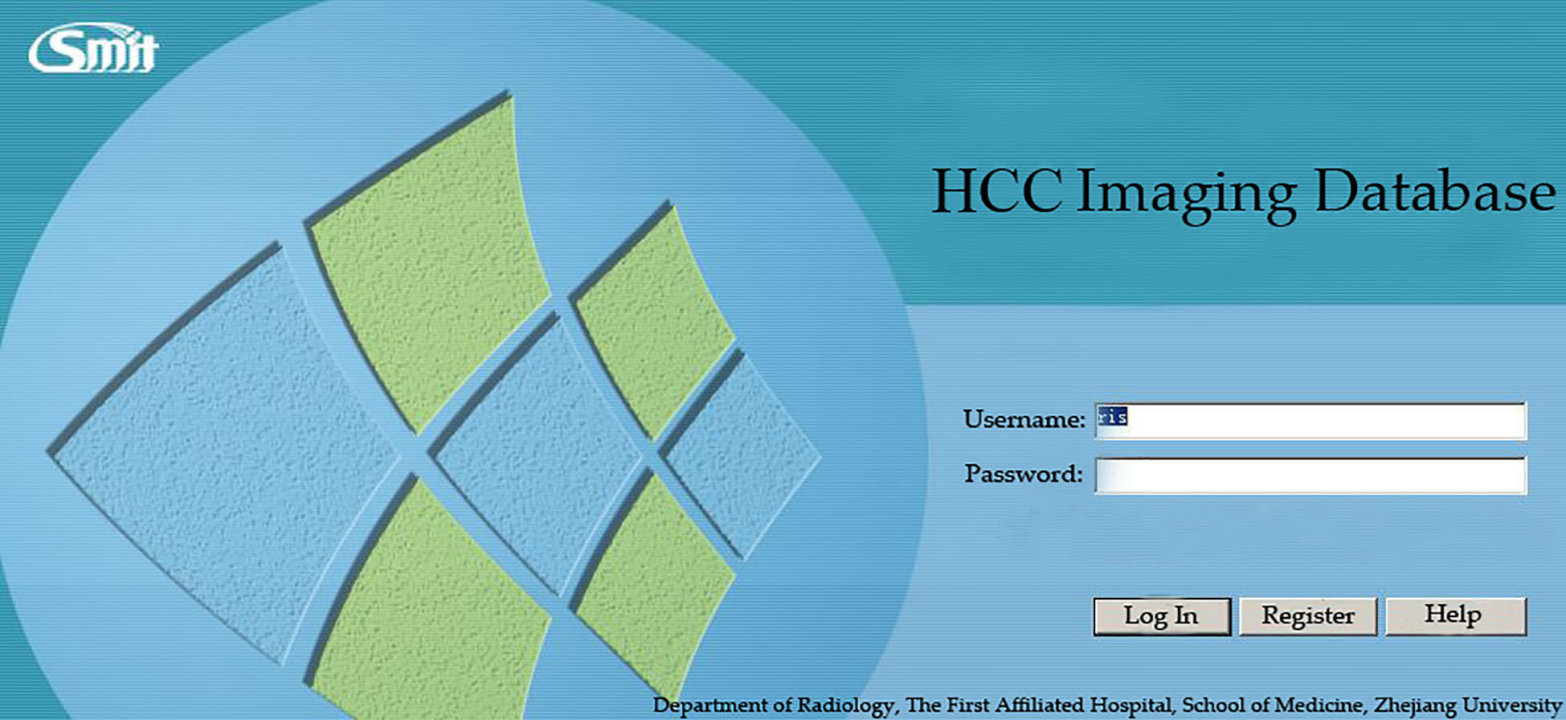
System login interface. HCC=hepatocellular carcinoma.

**Figure 4:**
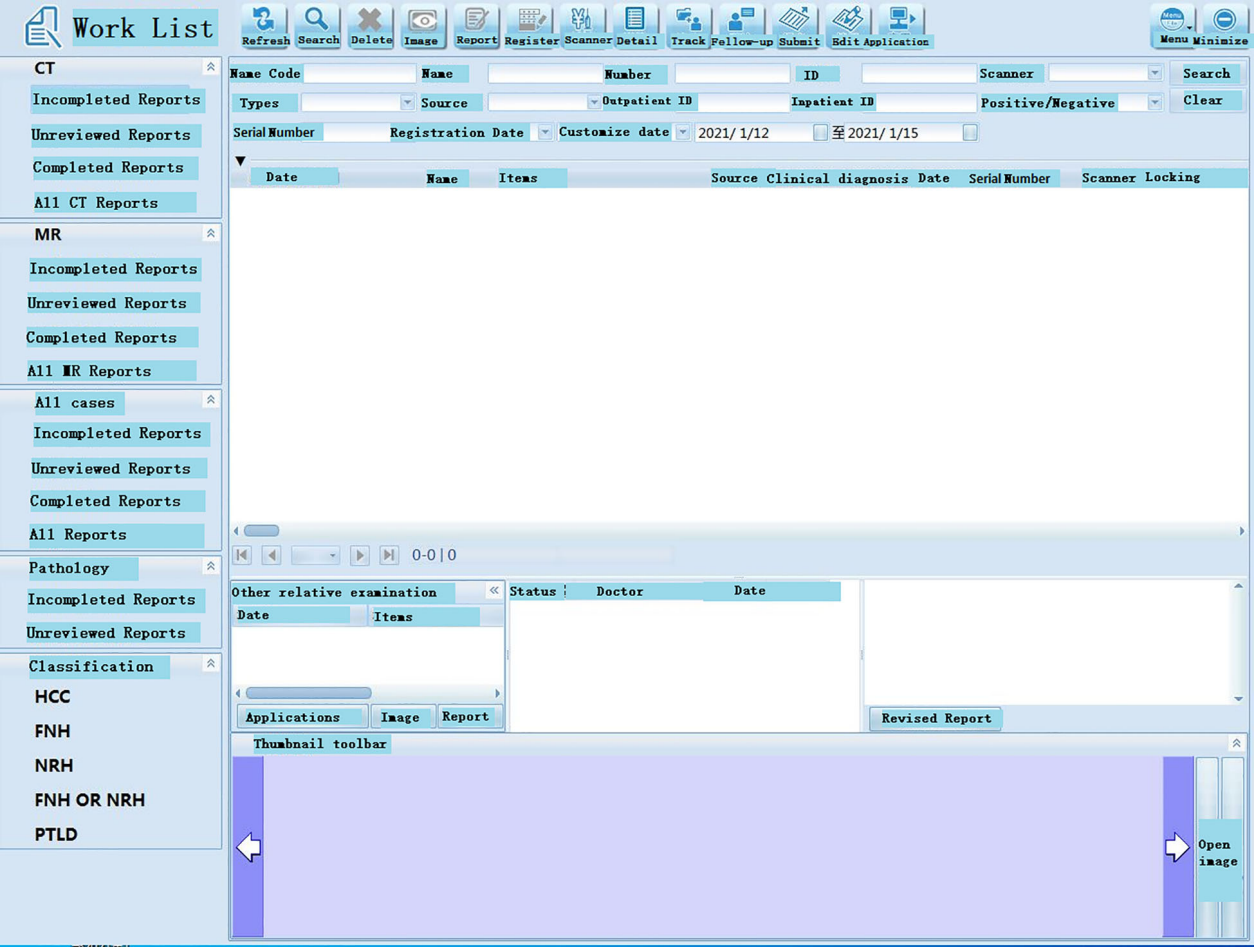
Working interface of the software. Database indexes are used for faster querying. In the system, patient’s ID number, gender, age, and date of admission and discharge are defined as a database index.

**Figure 5:**
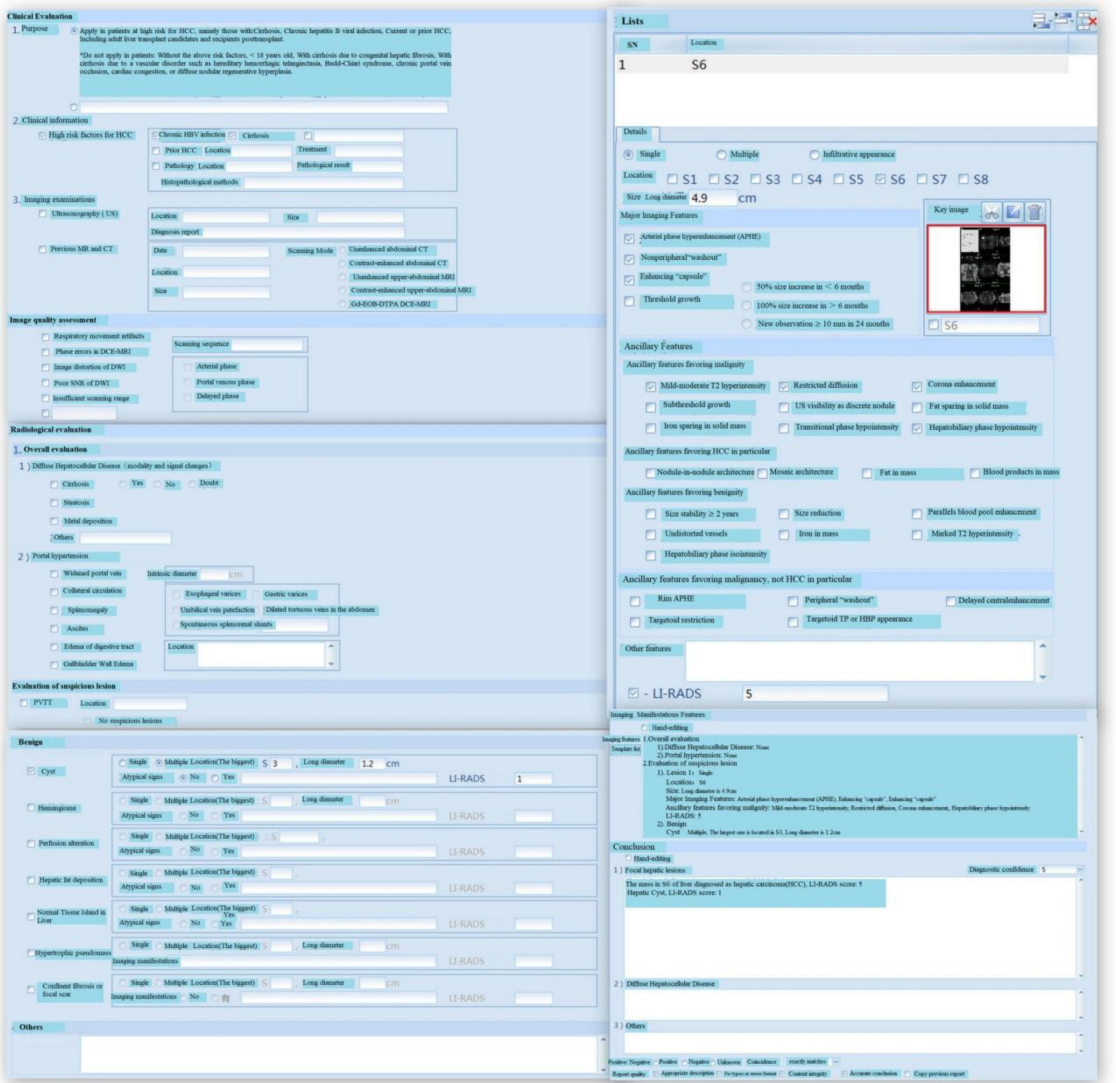
LI-RADS and Case Analysis. HBV, Hepatitis B Virus; Gd-EOB-DTPA, Gadolinium-Ethoxybenzyl-Diethylenetriamine penta-acetic acid; DCE, Dynamic Contrast Enhanced; DWI, Diffusion Weighted Imaging; SNR, Signal-to-Noise Ratio; PVTT, Portal Vein Tumor Thrombosis; HBP, Hepatobiliary Phase. LI-RADS is effective assessment tools for HCC and adopted by many clinical practices throughout the world. The score of LI-RADS can be calculated by filling in relevant contents according to the form.

**Figure 6:**
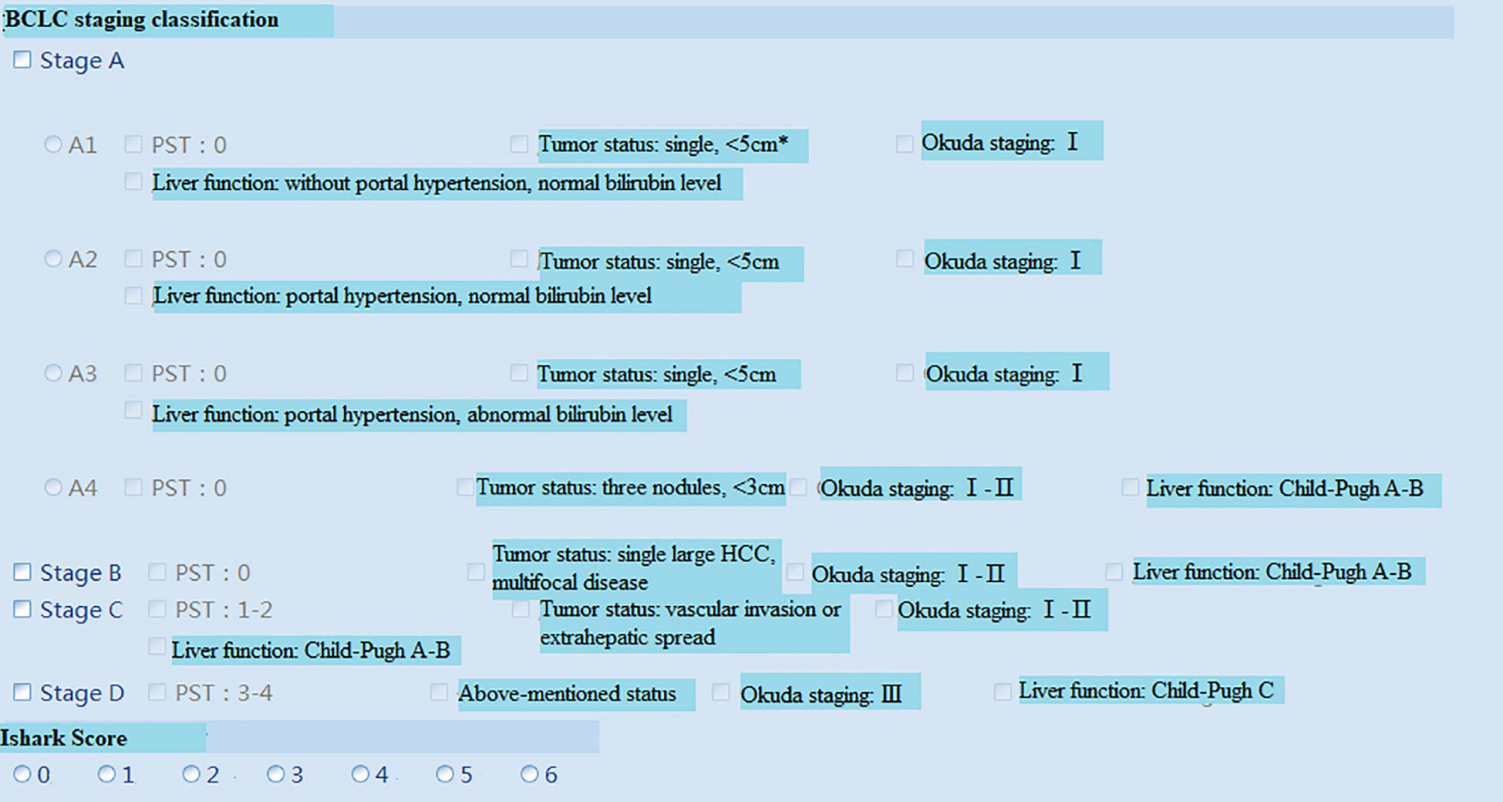
BCLC staging system[14]. *The size of a single tumor is not stated in the BCLC staging system, but tumor size is an influence factor for transplant selection, radiofrequency ablation (RFA) and surgical resection. Hence, the inclusion criteria of single HCC<5cm based on the reference[14]. BCLC, Barcelona Clinic Liver Cancer; PST, Performance Status Test.

**Figure 7:**
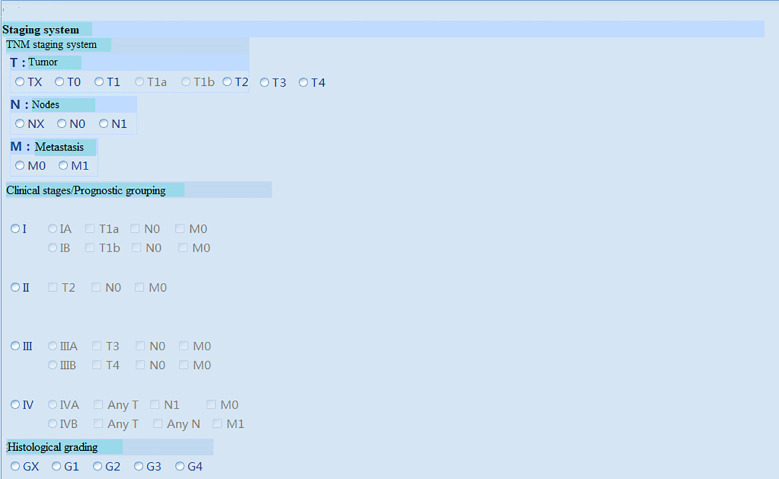
TNM staging system.

**Figure 8:**
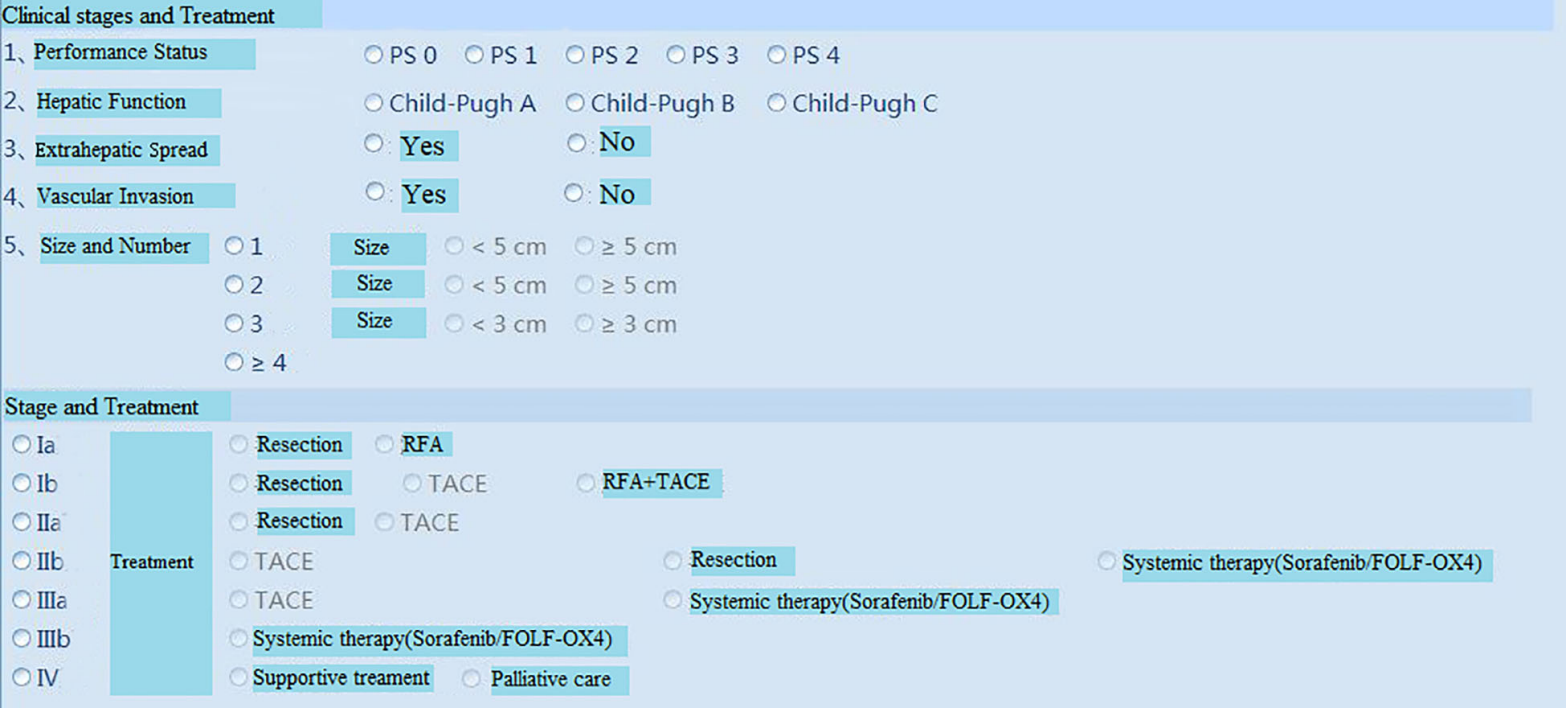
Clinical stages and Treatment roadmap. RFA=radiofrequency ablation.

**Figure 9:**
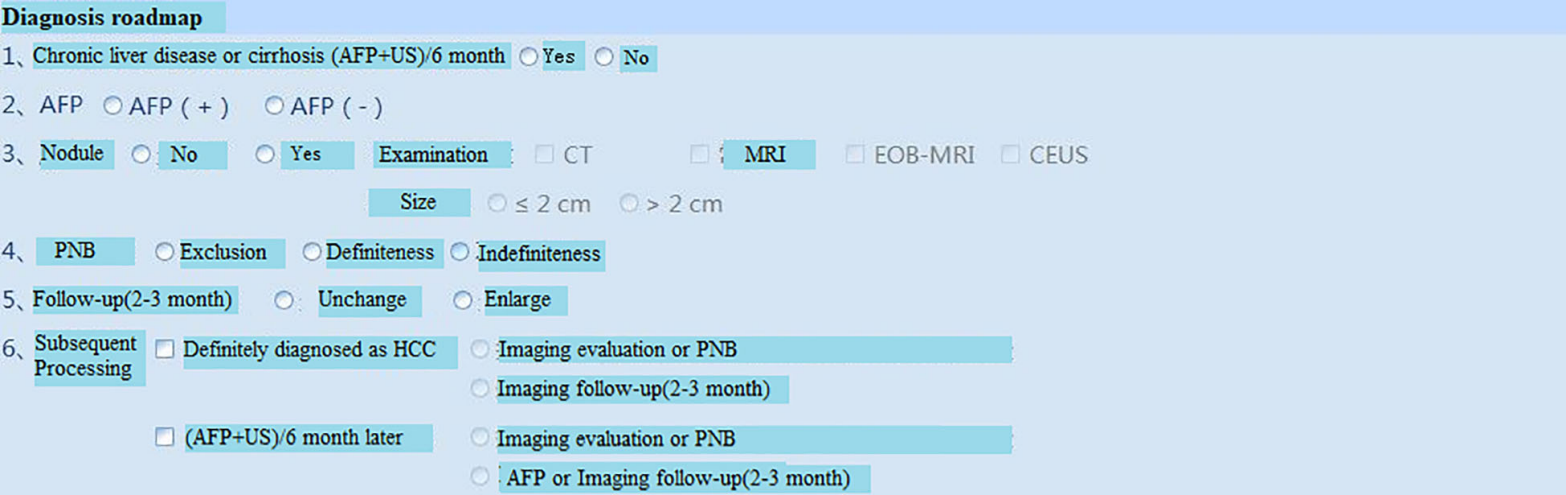
Diagnosis roadmap. PNB, Percutaneous needle biopsy.

The proposed system was tested, and the client, under the Windows operating system in the local area network of the hospital, could successfully login into the system (**Figure 3** (software login interface)). After searching through keywords, relevant cases could be inquired, sorted, and summarized (**Figure 4** search interfaces), and then, quantitative assessment and clinical treatment guidance could be performed on the data of the target cases, including various series of assessments (**Figure 5** (LI-RADS), **Figure 6** (BCLC), **Figure 7** (TNM classification)), clinical staging (**Figure 8**), treatment roadmap (**Figure 8**), diagnostic diagram (**Figure 9**), etc. The results showed that the system could successfully realize the management of HCC cases scientifically in the local area network of the hospital, and effectively ensure the data security and consistency as well.

## 4 Discussion

PACS and HIS have provided great improvements in the field of health services, the HCC data have a vital importance for diagnosis and treatment. However, HIS have brought along plenty problems as well as the amount of data is increasing day by day. It is simply enough to integrate the various data sources of patients, the medical faculty spends a lot of time to analyze these scattered information data. A comprehensive database which include developing computer-aided detection (CAD) or diagnosis methods is an effective tool to save time. Our present work has demonstrated the establishment of a framework of HCC imaging database and the system has only undergone preliminary testing. The outcome is promising as indicated in the results. The HCC imaging database can not only provide a large number of imaging data sources for the basic and clinical research of HCC, but also facilitate the scientific management and quality control of HCC, including the personalized treatment and follow-up of HCC patients. Therefore, we intend to use the platform in two aspects in the near future. One is to use it in our daily clinical work of HCC, providing a one-stop shop structured imaging report of HCC as explained above, another is to use it to support our clinical research of HCC, focusing on the imaging work.

Since the system is only used in local area network of the hospital, using the C/S architecture can guarantee the system stability and security, the response speed is also faster, furthermore it is suited for high throughput caching and background data processing. In addition, the C/S architecture can reduce the dependency between layers, rationally structured, highly cohesive, loosely coupled, easy to maintain and extend. The SQL Server is a good match for the C/S architecture, which is open source, excellent performance and great portability. Moreover, it is drastically improves execution time reviews, and is an effective management of medical picture archiving and filing. These new structures have the advantages of simple convenient, reliable operation, and quick access for management.

We demonstrated that the tool is able to quickly search the pathological, clinical and imaging data of HCC from PACS and HIS and perform some simple statistic analysis of HCC characteristics for further exploration. Furthermore, some intelligent diagnostic procedures can be implemented *via* the repository of the database, including the LI-RADS grading, HCC staging according to domestic and international guideline, and finally, a one-stop structured imaging report of HCC can be generated based on the database, including the imaging diagnosis, LI-RADS grading, staging and even a proposed treatment measure of HCC. Thus, the clinical decision making and management of HCC may be improved efficiently.

## 5 Limitations

There are several limitations to our study. First, our present work has demonstrated the establishment of a framework of HCC imaging database and the system has only undergone preliminary testing. The practical application in clinical work is our direction for the future work. Second, although it is an effective tool to save a lot of time in the assessment of HCC using our database, but efficient and effective use of the HCC data still affected by several barriers. Especially, after the target cases are collected in the database, the software performs quantitative assessment and LI-RADS assessment which require human intervention, this progress will consume much time and manpower. We expect to get help from the developing computer-aided detection(CAD) in the course of further development. Third, Our database and software is only used within the LAN, it is limited for the beneficiaries, so we hope the further extension which will benefit for other medical institutions.

## 6 Conclusions

The establishment of a HCC imaging database is of great significance for data management, scientific research, and diagnosis and treatment of HCC. This study proposed the successful design and development of a HCC imaging database on the basis of the C/S architecture using a three-tier architecture model. The proposed database can be used for intelligent management of HCC imaging data, accurate imaging evaluation, and auxiliary support for clinical decisions. It can also provide a shared resource and a reliable management platform for clinical testing, teaching, and scientific research on HCC.

## Data availability statement

The original contributions presented in the study are included in the article/supplementary materials, further inquiries can be directed to the corresponding author/s.

## Author contributions

Guarantor of integrity of the entire study: FC. Study concepts and design: YS, YT. Literature research: YS, JL. Clinical studies: YS, YT. Experimental studies/data analysis: RZ, YS, ZL. Statistical analysis: YS, JL, ZL. Manuscript preparation: YS, YT. Manuscript editing: YS, JL, FC. All authors contributed to the article and approved the submitted version.
